# Experiences of human immunodeficiency virus-positive postpartum women in Angola: Challenges and opportunities in preventing vertical transmission

**DOI:** 10.4102/phcfm.v18i1.5471

**Published:** 2026-07-23

**Authors:** Paulo N. Solari, Joana C. Pires, Pedro M. Aguiar, Ana R. Goes, Gonçalo F. Augusto

**Affiliations:** 1 Public Health Research Centre; Comprehensive Health Research Center, CHRC, LA-REAL, CCAL, NOVA National School of Public Health, NOVA University Lisbon, Lisbon, Portugal; 2 National Institute for Health Research of Angola (INIS), Luanda, Angola

**Keywords:** HIV, PMTCT, postpartum, qualitative research, maternal health, sub-Saharan Africa

## Abstract

**Background:**

Despite advances in the prevention of mother-to-child transmission (PMTCT) of human immunodeficiency virus (HIV), challenges persist in retention in care, antiretroviral therapy (ART) adherence and follow-up of HIV-exposed infants, particularly postpartum. In Angola, qualitative evidence on women’s lived experiences remains limited.

**Aim:**

To explore the experiences of HIV-positive postpartum women and identify opportunities to strengthen PMTCT services in Angola.

**Setting:**

The study was conducted at Lucrécia Paim Maternity Hospital, a major maternal and child health referral centre in Luanda, Angola.

**Methods:**

A qualitative study with an experiential orientation informed by phenomenological sensitivity was conducted between April 2025 and July 2025. Semi-structured interviews were conducted with 20 women up to 6 months postpartum, recruited through purposeful sampling from institutional records. Interviews were transcribed verbatim and analysed using reflexive thematic analysis following Braun and Clarke, supported by qualitative data analysis (MAXQDA) software.

**Results:**

Four themes emerged: discovery and acceptance of diagnosis, marked by emotional distress and gradual adaptation; pregnancy and childbirth, characterised by supportive care and episodes of stigmatisation; postpartum and baby care, involving heightened responsibility and challenges with medication and infant feeding; and infant testing, experienced with anxiety until confirmation of serological status. Post hoc comparisons by age group and partner status suggested variations in psychosocial needs and care experiences.

**Conclusion:**

Postpartum PMTCT is influenced by emotional, relational and institutional determinants affecting continuity of care. Strengthening integrated, woman-centred models with psychosocial support, consistent communication and stigma reduction is essential.

**Contribution:**

This study provides context-specific qualitative evidence to improve postpartum PMTCT services in Angola and similar settings.

## Introduction

Despite major progress in human immunodeficiency virus (HIV) prevention and treatment, HIV remains a global public health challenge. In 2023, an estimated 39.9 million people were living with HIV, with 1.3 million new infections and 63 000 acquired immunodeficiency syndrome (AIDS)-related deaths, highlighting persistent gaps in prevention, treatment and continuity of care, including in maternal and child health.^1^ Option B+, introduced in 2011–2012, recommends immediate and lifelong antiretroviral therapy (ART) for all pregnant and breastfeeding women living with HIV, regardless of cluster of differentiation 4 (CD4) count or clinical stage. It aims to reduce mother-to-child transmission, improve maternal health and simplify the prevention of mother-to-child transmission (PMTCT) of HIV service delivery.^2^ In African settings, retention in HIV care during pregnancy and postpartum remains challenging in the Option B+ era; a systematic review estimated 12-month retention at 76.4%, with particular vulnerability after delivery.^3^ Quality improvement initiatives such as process mapping and quality improvement (PROMAQI) may support monitoring of engagement and follow-up in Option B+ programmes.^4^ Current World Health Organization (WHO) guidance no longer uses the designation ‘Option B+’ but reinforces its core principle by recommending ART for all people living with HIV, including pregnant and breastfeeding women, and differentiated service delivery to support retention and adherence.^5^ World Health Organization and United Nations Children’s Fund (UNICEF) also recommend breastfeeding for at least 12 months, and up to 24 months or longer, when effective ART and adherence support are sustained,^6,7^ although women living with HIV in sub-Saharan Africa tend to breastfeed for shorter durations than HIV-negative women.^8^ Antiretroviral therapy initiation, adherence and retention among pregnant and postpartum women living with HIV are shaped by individual and contextual factors, including knowledge of HIV and PMTCT, treatment demands, disclosure and partner involvement.^9^ Qualitative evidence from Ethiopia also shows that stigma, adverse marital dynamics, economic barriers, religious beliefs and confidentiality concerns contribute to loss to follow-up, whereas peer support, partner involvement and active tracing may promote re-engagement in care.^10^ These dimensions make comparisons by age (< 25 vs ≥ 25 years) and by partner partner support and involvement (present vs absent) relevant, given that younger women are at greater risk of loss to follow-up and partner support and involvement is associated with greater adherence and retention in PMTCT programmes.^11,12,13^ In Angola, HIV remains a relevant public health challenge, with UNAIDS reporting an estimated national prevalence of 1.5%, approximately 310 000 people living with HIV and 15 000 new infections in 2022. Pregnant women remain a vulnerable group, with sentinel surveillance reporting HIV prevalence of 2.2% among women aged 15–49 years. Despite progress, ART coverage in 2022 remained below global targets, estimated at 49% among adults and 22% among children living with HIV.^14^ The National HIV/AIDS Strategic Plan 2023–2026 seeks to expand HIV prevention, testing and treatment, including efforts to strengthen PMTCT services, follow-up of HIV-exposed children and use of vertical transmission data.^15^ Previous Angola-based studies have highlighted persistent challenges in PMTCT service use and programme effectiveness, reinforcing the need to strengthen access, continuity of maternal–child HIV care and follow-up of HIV-exposed children.^16,17^ Incomplete knowledge of HIV and AIDS among Angolans aged 15–49 years further supports the need for context-sensitive counselling and communication.^18^ However, qualitative evidence on women’s postpartum experiences remains limited, despite this being a critical period for ART adherence, early infant diagnosis (EID), infant feeding decisions and prevention of loss to follow-up along the PMTCT continuum.^19,20^ This qualitative study, with an experiential orientation informed by phenomenological sensitivity, explored the lived experiences of HIV-positive postpartum women at Lucrécia Paim Maternity Hospital, Luanda, Angola and identified opportunities to strengthen postpartum PMTCT services. Comparisons by age and partner status examined variations in psychosocial needs and care experiences across groups.

## Research methods and design

### Study design

This was not designed as an interpretative phenomenological study. Rather, it was a qualitative study situated within an interpretivist and contextualist paradigm, with an experiential orientation informed by phenomenological sensitivity. The study sought to understand participants’ lived postpartum experiences without conducting a formal phenomenological analysis. Reflexive thematic analysis, following Braun and Clarke, was used to identify patterns of meaning grounded in participants’ accounts, rather than to apply interpretative phenomenological analysis or Giorgi’s descriptive phenomenological method.

### Setting

The study was conducted between April 2025 and July 2025 at Lucrécia Paim Maternity Hospital, a major public referral maternity hospital in Luanda, Angola. The hospital provides antenatal, delivery and postnatal care, including PMTCT services for pregnant and postpartum women living with HIV. These services include HIV counselling and follow-up, ART adherence support, infant prophylaxis, infant feeding counselling and referral or follow-up for early infant diagnosis. The hospital serves women from Luanda and surrounding urban and peri-urban communities with diverse socioeconomic conditions and variable access to transport, family support and continuity of care. Portuguese, the official language and main language of clinical care, was used in the interviews, although national languages such as Kimbundu and Umbundu are also spoken in Luanda. Participant ethnicity was not collected and was not used analytically.

### Population and sample

The target population comprised HIV-positive women who had given birth at Lucrécia Paim Maternity Hospital and were up to 6 months postpartum. Eligible women were identified from institutional records and contacted by telephone. Inclusion criteria were HIV diagnosis before or during the current pregnancy, delivery at the maternity hospital, postpartum status up to 6 months and follow-up in PMTCT services. Women were excluded if physical or mental conditions made the interview unfeasible or if they refused informed consent. Participants were recruited through purposeful sampling, guided by information power, until sufficient depth and diversity of experiences relevant to the research question had been achieved.^21^ Data sufficiency was considered adequate because the final interviews did not generate new analytically relevant codes or insights. Of 50 women contacted, 20 agreed to participate and completed the interview. Reasons for non-participation included non-response to telephone contact, explicit refusal and logistical difficulties. Some women also mentioned a lack of partner support or partner-related difficulties although these were not systematically quantified. Age group and partner status were not used as a priori sampling strata. Instead, they were extracted after recruitment and used as analytic comparison dimensions to explore variations in experiences by age (< 25 years vs ≥ 25 years) and partner status. ‘With partner’ referred to having a current partner recognised by the participant, regardless of cohabitation; ‘without partner’ indicated absence of a partner in the peripartum period.

### Data collection techniques

Data were collected through semi-structured individual interviews conducted in Portuguese, in a private room at the maternity ward, by one member of the research team, who was not part of the participants’ routine clinical care. Interviews lasted approximately 30 min – 60 min, allowed short pauses when participants became emotionally distressed, were audio recorded with consent and were supplemented by field notes on contextual and non-verbal observations. No non-participants were present, and no repeat interviews were conducted. The interview guide was developed from the study objectives and the PMTCT care pathway, covering HIV diagnosis and acceptance, pregnancy and childbirth, postpartum and baby care, ART adherence, infant feeding, infant testing, disclosure, social and family support and experiences with health professionals and services. The opening substantive question was: ‘Can you tell me how you found out that you have HIV?’ The full guide is provided as Online Appendix 1.

### Reflexivity

The research team included clinicians, epidemiologists and public health researchers with experience in HIV, maternal and child health and qualitative research. The interviewer was not a member of the participants’ routine care team, and contact occurred only during recruitment and interview. The team recognised that familiarity with PMTCT services in Angola could shape assumptions about stigma, service barriers and continuity of care.^22^ Reflexive discussions, analytic memos and repeated checking against transcripts were used to keep interpretations grounded in participants’ accounts.

### Data analysis

The audio-recorded interviews were transcribed verbatim in Portuguese and pseudonymised using alphanumeric codes. Transcripts were not returned to participants, and participant checking was not conducted. Quotations were translated into English by the research team and checked against the original Portuguese transcripts to preserve meaning and participant voice. Data were analysed using primarily inductive and semantic reflexive thematic analysis, following Braun and Clarke,^23,24,25^ with data management and coding performed in MAXQDA 2022.^26^ Following Braun and Clarke, the analysis proceeded through iterative phases. Firstly, all transcripts were read immersively to support deep familiarisation with the data. Secondly, line-by-line coding was conducted to capture participants’ meanings. Thirdly, codes were reviewed to remove redundancies, merge semantically similar codes and organise them into subcategories. Fourthly, analytic memos and interpretive summaries supported the development of candidate themes. Fifthly, themes and subthemes were reviewed and refined through constant comparison, with attention to recurring patterns, contrasts and negative cases. Sixthly, the thematic structure was defined, and representative quotations were selected to illustrate each theme.

### Trustworthiness

Trustworthiness was addressed through credibility, dependability, transferability, confirmability and reflexivity. Credibility and confirmability were supported by repeated reading of transcripts, comparison between codes and transcript excerpts, representative quotations, analytic memos and reflexive team discussions. Field notes were used to contextualise interviews and relevant non-verbal observations, not as a separate source for data triangulation. Dependability was supported by an audit trail of code lists, memos and decisions made during code refinement and theme development. Transferability was enhanced through a detailed description of the setting, recruitment process, participant characteristics and care context. Coding and theme development followed a reflexive collaborative process within the five-member research team. Initial coding was undertaken by one researcher and reviewed with a second researcher; a third researcher reviewed the coding structure and its alignment with transcript excerpts. The evolving code list, analytic memos and candidate themes were then discussed by the full team. Differences in interpretation were not treated as inter-coder reliability disagreements but as opportunities for reflexive discussion and refinement of the thematic structure.

### Ethical considerations

Data confidentiality was ensured through pseudonymisation with alphanumeric codes, removal of direct identifiers from transcripts and secure password-protected storage of audio files and transcripts. The key linking participant identities to codes was stored separately with restricted access. Participation was voluntary, with the right to withdraw at any time without affecting clinical care, and all participants provided written informed consent before the interviews. The study was approved by the Ethics Committee of the Angolan Ministry of Health (Opinion No. 018/CEMS/2025, dated 05 May 2025). Reporting was guided by the Consolidated Criteria for Reporting Qualitative Research (COREQ),^27^ and the completed checklist is provided as Online Appendix 2.

## Results

The characteristics of the participants included in the study are presented in Table 1.

**TABLE 1:** Participant characteristics.

Participant	Age group (years)	Partner status	Postpartum interval at interview (months)	Timing of HIV diagnosis	ART status at interview
P1	≥ 25	With partner	2–6	During pregnancy	On ART
P2	≥ 25	With partner	2–6	Timing unclear	On ART
P3	< 25	Without partner	2–6	Timing unclear	On ART
P4	< 25	Without partner	2–6	During pregnancy	On ART
P5	< 25	Without partner	2–6	During pregnancy	On ART
P6	≥ 25	With partner	2–6	During pregnancy	On ART
P7	≥ 25	With partner	2–6	During pregnancy	On ART
P8	< 25	With partner	2–6	Before current pregnancy	On ART
P9	≥ 25	With partner	≤ 1	During pregnancy	On ART
P10	< 25	Without partner	2–6	During pregnancy	Not on ART at interview
P11	≥ 25	Without partner	≤ 1	Before current pregnancy	On ART
P12	≥ 25	With partner	≤ 1	During pregnancy	On ART
P13	≥ 25	With partner	≤ 1	Before current pregnancy	On ART
P14	≥ 25	With partner	2–6	Before pregnancy	On ART
P15	≥ 25	With partner	≤ 1	During pregnancy	On ART
P16	≥ 25	With partner	≤ 1	Before current pregnancy	On ART
P17	≥ 25	With partner	≤ 1	During pregnancy	On ART
P18	≥ 25	With partner	≤ 1	During pregnancy	On ART
P19	< 25	With partner	≤ 1	During pregnancy	On ART
P20	≥ 25	With partner	≤ 1	During first pregnancy	On ART

HIV, human immunodeficiency virus; ART, antiretroviral therapy.

Twenty HIV-positive women participated in the study, identified through institutional records of women who gave birth at the Lucrécia Paim Maternity Hospital in the 6 months prior to data collection. At the time of the interview, all were in the postpartum period (≤ 6 months after delivery). Ages were grouped into two strata: < 25 years (*n* = 6) and ≥ 25 years (*n* = 14). Regarding partner status, most participants had a partner (*n* = 15), while five were without a partner. Regarding ART status, 19 participants were on ART at the time of the interview, while one participant, P10, was not on ART. Her account was therefore interpreted as an individual illustration of discontinuity of care and counselling gaps across the postpartum PMTCT pathway. Analysis of the interviews identified four interconnected themes reflecting how women negotiated the emotional, relational and institutional dimensions of living with HIV during the postpartum period. These themes addressed the discovery and acceptance of diagnosis, experiences during pregnancy and childbirth, postpartum and baby care and experiences related to infant testing and follow-up.

### Discovery and acceptance of the diagnosis

The discovery of the HIV diagnosis was a significant and multifaceted event in the lives of the women interviewed, involving complex emotional, social and spiritual dimensions. The process of acceptance emerged gradually and non-linearly, interspersed with moments of denial, suffering, resignation and, in many cases, overcoming and rebuilding identity. The circumstances in which the participants learned of their diagnosis varied. For several women, the diagnosis occurred during pregnancy or at the time of delivery: ‘I found out I was HIV-positive when I was 3 months pregnant’ (P1); Others reported the discovery during routine examinations or in the context of other health care, ‘when I went for a routine consultation’ (P2), sometimes associated with non-specific symptoms ‘I had rashes in the vaginal area’ (P3). In some cases, the diagnosis resulted from voluntary rapid HIV testing. Initial reactions were often marked by intense psychological distress, despair and existential questioning, ‘Why me, God?’ (P18), including thoughts of death ‘I thought about dying instead of living’ (P16) and ‘I wanted a car to come and hit me’ (P5). Fear and uncertainty were strongly associated with pregnancy and the baby’s well-being, with recurring concerns about the possibility of vertical transmission: ‘Will my child be born with it?’ (P4). A comparison by age group suggested different emotional trajectories following an HIV diagnosis. Among younger women (< 25 years), the diagnosis was more frequently experienced as an abrupt rupture of identity and future expectations, marked by shock, despair and emotional instability: ‘I’m still a child … the person who did this ruined my life’ (P8); ‘At that moment, I wanted to die’ (P5). In contrast, women aged ≥ 25 years more frequently described the diagnosis within trajectories already shaped by motherhood and family responsibilities, with narratives of gradual adaptation and practical acceptance of the condition: ‘I couldn’t do anything else but follow the treatment’ (P11); ‘I looked at my children and saw that life didn’t end there’ (P15). Differences also emerged according to partner status, albeit in a heterogeneous manner. Some women with partners reported significant emotional support, such as ‘It was my partner who was giving me strength’ (P6), while others described fear associated with revealing the diagnosis: ‘My husband doesn’t know … I thought it wouldn’t be easy for him’ (P13). Among women without a partner, coping was most often supported by mothers or sisters: ‘My sister was the one who gave me strength’ (P5). Acceptance of the diagnosis emerged as a gradual and emotionally negotiated process, often marked by initial resistance and disbelief: ‘At first I thought it was a lie’ (P4). Motherhood subsequently became a central framework through which participants reinterpreted their diagnosis and reconstructed their sense of purpose. The bond with their children was described as decisive in transforming the diagnosis from an experience associated with fear and hopelessness into a motivation for treatment adherence and future planning: ‘I can only accept this through my baby’ (P12); ‘Life wasn’t over’ (P13); ‘My children need me’ (P19). Contact with health services emerged as a factor facilitating acceptance, especially through information about the existence of treatment and the relational support provided by professionals, ‘They said there was a treatment’ (P4) and ‘The doctor talked to me a lot’ (P9). Some participants also mentioned actively seeking information as a way of dealing with their initial fear, ‘I watched some videos on the internet’ (P4), as well as a process of gradually getting used to the diagnosis, ‘I got used to it’ (P7), accompanied, in some cases, by a critical reflection on stigma, ‘We should not discriminate against those who have this condition’ (P16). With regard to disclosing the diagnosis, the participants described selective and judicious choices, favouring close family members and health professionals. For some, sharing was restricted to a very limited circle, ‘just me, the doctor and my husband’ (P15), while others chose not to disclose it to their partner, sharing the information with other family members or trusted friends, ‘I spoke to my aunt’ (P8); ‘she is a trustworthy person’ (P14). Some women reported keeping the diagnosis confidential as a strategy for emotional protection, ‘I endured everything alone’ (P11), while others experienced disclosure as a relief, ‘a weight was lifted off my shoulders’ (P14). The fear of revealing the diagnosis was strongly associated with the fear of judgement, rejection and discrimination: ‘She is the kind of person who discriminates’ (P17); ‘My biggest fear is being judged’ (P10). These fears were contextualised in a social environment marked by persistent stigma associated with HIV: ‘Here in Angola, this disease is still very taboo’ (P16). The support of health professionals, partners and family members was identified as a mitigating factor in the initial suffering. In the case of professionals, this support included encouragement, explanations and counselling, ‘they were encouraging’ (P2); ‘they told me not to give up’ (P16). The start of treatment was described by some participants as a moment of psychological tension, marked by the difficulty in accepting the need for continuous medication, ‘the hardest thing was accepting the idea that I would have to take it forever’ (P13), and by the requirement for strict schedules, ‘we have to choose a time’ (P19). Difficulties related to adverse effects and occasional shortages of medication were also mentioned, experienced as a source of anxiety, ‘sometimes it is difficult to get’ (P1). Despite these constraints, most participants described stable and disciplined adherence to treatment, integrating medication into their daily routine, ‘I take it every day at 9 p.m.’ (P20).

### Pregnancy and childbirth

The experience of pregnancy and childbirth among women living with HIV proved to be a journey marked by emotional tensions, communication challenges and contrasting experiences of acceptance and respect in healthcare. Although most participants described clinical and obstetric care as generally satisfactory, narratives emerged that highlighted situations of emotional and symbolic vulnerability associated with stigma, fear of vertical transmission and structural limitations of the healthcare system.

Pregnancy was experienced under intense emotional strain, marked by anxiety, fear and hope.

The discovery of pregnancy intensified concerns related to the diagnosis, with some women expressing fears about their child’s future, questioning whether they ‘will go through what I am going through’ (P4) or stating that ‘just knowing I was pregnant worried me’ (P16). Some participants reported seeking psychological support during pregnancy, mentioning specialised consultations: ‘I decided to come to the Lucrécia Maternity Hospital to consult with psychologists’ (P12). Age-related differences also emerged in experiences relating to pregnancy and childbirth. Among younger women (< 25 years), concerns about vertical transmission and protecting the baby were particularly visible: ‘Will the baby I’m going to have also have this virus?’ (P4). Among women aged ≥ 25 years, narratives more often reflected accumulated maternal responsibilities and concerns about the quality and continuity of care, including dissatisfaction with services: ‘They didn’t see me; I left here very upset’ (P9). Partner status also shaped pregnancy experiences in complex ways. Some women with a partner described emotional support during pregnancy: ‘My partner gave me a lot of strength’ (P6), whereas others reported emotional distance or difficulties related to disclosing the diagnosis despite being in a relationship: ‘My husband doesn’t know’ (P13); ‘Since I got pregnant, that attention hasn’t been the same’ (P12). Among women without a partner, pregnancy could be experienced as emotionally lonely, as illustrated by one participant: ‘I am alone without my husband’ (P4). Most participants highlighted positive experiences of humanised care, referring to welcoming attitudes, empathy and respect on the part of the health teams throughout the obstetric follow-up and at the time of delivery. The absence of discriminatory language was particularly valued: ‘They never said “you have this or that”’ (P4). In contrast, some women reported isolated episodes of judgement and discrimination in a clinical context, as illustrated by P3: ‘A professional said: “you asked for it”. I felt judged.’ At the same time, narratives emerged of fragility in the monitoring during pregnancy, mainly associated with communication failures and inconsistencies in medical guidance, which generated confusion and insecurity. Some participants mentioned a lack of clear explanations about the care to be taken, ‘They just said I should be careful, but they didn’t explain it well’ (P10), while others reported contradictory messages regarding the type of delivery, ‘Some doctors said that a caesarean section was best, others said it wasn’t’ (P19). Childbirth was described as an intense and emotionally significant event. Some participants reported positive experiences, associated with feelings of protection and trust, ‘It went well, thank God. I felt protected, confident’ (P19) and valued interpersonal interactions with health professionals, highlighting gestures of attention and emotional care, ‘He talked to me, I felt supported’ (P4); ‘The doctor who stitched me up was patient and loving with me’ (P12). However, others reported experiences of insecurity and discomfort associated with clinical interventions that were poorly explained and experienced as invasive, which generated a feeling of loss of control over their own bodies, as illustrated by reports of procedures performed without prior explanation, ‘She gave me an injection, I screamed’ (P9) and ‘Defecate in the bucket’ (P12).

### Postpartum and baby care

The postpartum period was described as a time of profound emotional adjustment and reorganisation of daily life for women living with HIV. The birth of the baby marked a significant change in the experience of motherhood, with feelings of joy coexisting with the need to adjust to the new reality. The emotional impact of the first encounter was described as transformative: ‘I saw my baby on the floor, and everything changed’ (P14). The emotional bond with the baby emerged as the central axis of the postpartum experience, combining affection and maternal fulfilment with heightened vigilance, responsibility and persistent concern about the child’s well-being. ‘The diagnosis led to the adoption of extra care in daily life, expressed in constant concern for the child’s well-being, ‘a lot of responsibility and extra care’ (P14). This bond was described as the main source of motivation, associated with the desire to ‘create ties’ (P13) and ‘be an exemplary mother’ (P17). Postpartum daily life was characterised by an overlap of tasks and demands, combining intensive baby care, domestic responsibilities and constant vigilance. This burden was experienced as physically exhausting, mainly because of night-time interruptions, ‘waking up at night’ (P4), although for some participants, care was also a deeply rewarding experience, stating that it was something they ‘really wanted’ (P4). In daily care, mothers described the implementation of strict hygiene and disinfection routines, as well as the separation of personal items, reflecting constant vigilance regarding risk prevention and infant protection: ‘very careful with hygiene: The plate, the bottle, the clothes’ (P20). Managing the baby’s medication was a central aspect of the postpartum period. The participants demonstrated understanding and commitment to the baby’s therapy, reporting clear guidelines on daily administration, ‘they prescribed Bactrim: They said I have to give it every day’ (P4), and the importance of not missing medication, ‘I shouldn’t miss the medication’ (P5). Compliance with the treatment plan was associated with protecting the baby, ‘may it protect him’ (P13). Practical aspects of medication supply were also mentioned, such as the need to return to the hospital, ‘they gave me the syrup for the baby and asked me to come back’ (P14), as well as feelings of concern when seeing the baby medicated, ‘it worries me to see a baby already taking adult medication’ (P16). Paediatric follow-up was described as a source of relief and reaffirmation of hope, contributing to feelings of tranquillity and confidence about the child’s future. The participants associated this follow-up with the perception of good health and development of the baby, expressing satisfaction with the clinical evolution, as illustrated by expressions such as ‘everything has been going well’ (P8) and ‘he was born healthy and is developing normally’ (P14). The relationship with health professionals was experienced in an ambivalent way. Some participants reported clarity and confidence in the guidance received, ‘they explained what I should do’ (P16), and expressed high adherence to the recommendations, prioritising the child’s well-being, ‘I did everything that was recommended’ (P3). In contrast, others reported gaps in information, contradictions in the guidance and a lack of support, expressed in feelings of confusion, ‘they didn’t explain much’ (P10), and helplessness, ‘I never received help from anyone’ (P11). Persistent doubts arose, especially around breastfeeding and medication, ‘I didn’t know if it was for me or for her [*the medication*]’ (P10). During the postpartum period, age-related differences were less clear-cut, but variations were observed in the level of practical confidence with care routines. Among younger women (< 25 years), reports indicated greater reliance on professional guidance and uncertainty regarding the management of the baby’s feeding and medication: P4 described it as difficult to ‘wake up at night to formula milk’, while P10 mentioned a lack of guidance after the birth: ‘Did you receive any help or guidance after the baby was born?’ ‘No’ and doubts about medication: ‘I don’t know if it’s for me or for the baby’. Among women aged ≥ 25 years, care was more frequently integrated into already established maternal routines, albeit with close monitoring: ‘My treatment is with tablets … and with the babies it’s the same … there’s follow-up with syrup’ (P11). As for partner status, women without a partner tended to report greater individual responsibility, such as P11, who stated: ‘I’ve never received help from anyone’, while some women with a partner mentioned practical support, such as P6: ‘when I go to collect the medication, I have to leave the baby with the father’. However, the presence of a partner did not eliminate doubts or insecurities, as shown by P13: ‘My biggest concern is whether, as I’m breastfeeding my baby, she won’t have this problem’.

Breastfeeding emerged as one of the most sensitive topics in the postpartum period.

Some women reported choosing not to breastfeed, following advice to use formula milk, stating that ‘it is best not to breastfeed’ (P19). The decision was described as emotionally difficult, ‘the hardest thing was not breastfeeding, because I wanted to breastfeed my child’ (P3). Although the choice not to breastfeed was associated with protecting the baby, the underlying clinical rationale was not always explained in detail in the participants’ discourse, often appearing as medical advice followed based on trust in professionals. Difficulties in managing this decision socially were also mentioned, particularly within the family: ‘it was difficult to explain to my family why I wasn’t breastfeeding, they asked a lot of questions, and I didn’t really know what to say’ (P3). In contrast, there were reports that the guidance received from health professionals contributed to greater peace of mind and confidence in the decision, ‘the doctor explained it well and said it was better this way, which reassured me’ (P16). In less frequent cases, some participants reported following the guidance to breastfeed, ‘I decided to follow it’ (P13). The interviews did not allow us to determine whether these different infant-feeding recommendations reflected individual clinical considerations, local implementation of guidance or inconsistent counselling.

### Baby tests

The participants’ narratives reveal that testing the baby took on central significance in the postpartum period, being experienced as a time of high expectation, anxiety and vigilance. For many mothers, this process was associated with the need to strictly comply with the guidelines received, with a view to protecting the baby while awaiting the results. The moment of testing was described as a period of intense anxiety, centred on concern for the child’s health. After birth, the mothers’ focus shifted almost exclusively to the possibility of whether or not the baby was infected, which was the main cause of concern: ‘my biggest concern is that he gets rid of this’ (P11) and ‘whether or not she has the disease’ (P8). Experiences relating to testing the baby were marked by anxiety in both age groups, albeit with distinct nuances. Among younger women (< 25 years), testing was more closely associated with the fear of confirmation of transmission and the anticipation of maternal guilt: ‘That fear … will they say she’s a carrier of the disease?’ (P8); ‘I’ll blame myself a little for this’ (P8). Among women aged ≥ 25 years, anxiety was also present but was more frequently framed within the context of clinical follow-up and waiting for the final result: ‘I’m anxious to see my daughter’s result’ (P7); ‘Every mother has that worry; only after getting the final test result does the mother feel at ease’ (P17). As for partner status, the differences were less marked: Having a partner did not eliminate anxiety but may have encouraged greater emotional sharing, as in P8, who stated that ‘my daughter and my husband’ were her motivation; among women without a partner, concern appeared more closely linked to individual responsibility for care, as in P10, who summed up her concern as ‘looking after’ her daughter. The interval between the test and the communication of the result was experienced as a time of fear and uncertainty. Some participants reported nervousness associated with previous behaviours and the possible consequences for the baby, ‘I was nervous because I breastfed for three weeks’ (P9). Anxiety was exacerbated by the delay in communicating the results and the lack of clear information about deadlines, as expressed by P4, ‘come back later,’ and by P19, ‘they said only after six months.’ The moment the result was communicated was predominantly described as liberating, accompanied by feelings of relief, happiness and gratitude, ‘I was relieved: The result was negative’ (P4) and ‘very happy, because thank God the result was negative’ (P14). In several narratives, the wait and the test result were interpreted in the light of faith and spirituality, emerging as a source of hope during the period of uncertainty and as a form of relief after obtaining the result, ‘I went to thank God’ (P3) and ‘I trust in God’ (P12). Some participants also expressed satisfaction with the follow-up they received, stating that ‘they did some interventions and everything went well’ (P8). The family context emerged as a source of tension during this period, mainly associated with misunderstanding and fear on the part of other family members, which translated into frequent questions such as ‘why does the child have to do [*prophylaxis*]?’ (P8).

## Discussion

The central interpretation of this study is that postpartum PMTCT vulnerability should not be understood only as a biomedical issue of ART adherence, infant feeding or infant testing, but as a relational and organisational process shaped by stigma, disclosure, partner support, clinical communication and trust in health services. This interpretation is consistent with evidence showing that retention and adherence in PMTCT/Option B+ programmes are influenced by disclosure, social support, stigma and the quality-of-care experiences.^9,13,28,29^ In this Angolan referral setting, the findings suggest that strengthening postpartum PMTCT requires integrated mother–baby follow-up combined with psychosocial support, safe disclosure counselling and stigma-sensitive communication. These findings should also be interpreted within Angola’s current HIV policy context. The National HIV/AIDS Strategic Plan 2023–2026 aims to scale up HIV prevention, testing and treatment services while strengthening prevention of vertical transmission, follow-up of HIV-exposed children and the use of programme data. It is also framed by human rights and equity principles, with attention to structural barriers, stigma and discrimination, gender-based violence and community engagement in PMTCT, ART and data reporting systems.^14,15^ In this context, the participants’ accounts of stigma, inconsistent counselling, transport and economic constraints, partner-related barriers and uncertainty around infant follow-up suggest that strengthening postpartum PMTCT in Angola requires not only clinical monitoring but also better integration with primary health care, stigma-sensitive communication, community support and reliable mother–baby follow-up systems. The narratives reveal that an HIV diagnosis, especially when it occurs during pregnancy or peripartum, can be experienced as a biographical rupture, with fear, guilt, self-stigmatisation and intense anxiety about the baby’s future. Similar findings are described by Worku et al.,^30^ in which women with HIV report marked emotional distress during pregnancy and the postpartum period and a need for structured psychosocial support. In this sense, the results point to the importance of integrating mental health interventions (depression/anxiety screening, brief counselling and referral) into maternal and childcare and HIV follow-up, particularly in the immediate postpartum period. Ackerman et al. suggest that psychosocial interventions (provided by non-specialists with supervision) can improve mental health outcomes in rural/resource-limited settings.^31^ A key contribution of the study is to show that disclosure of diagnosis is not an ‘event’, but a process negotiated under social risk, including fear of abandonment, judgement, violence, loss of support and marital breakdown. This is consistent with evidence from sub-Saharan Africa showing that disclosure among pregnant and postpartum women remains challenging, and that HIV-related stigma can hinder disclosure, service uptake, adherence and continued engagement in PMTCT care.^28,29^ Qualitative literature in African contexts describes similar patterns, in which stigma (anticipated and experienced) conditions disclosure, the search for care and continued participation in vertical transmission prevention programmes.^32^ Additionally, a recent meta-analysis on adherence to Option B+ in sub-Saharan Africa identifies lack of disclosure and poor social support as factors consistently associated with non-adherence, reinforcing the programmatic reading that safe disclosure and social support are critical determinants.^13^ Thus, the results support the need for clinical strategies to support disclosure (individualised plans, step-by-step counselling, violence risk assessment) and models of partner involvement focused on women’s safety and autonomy, avoiding coercive approaches. Participants describe practical challenges (family routines, travel costs and time, adverse effects) but also less visible social mechanisms such as stigma at home and in the community, which amplify the risk of ART interruptions. This combination of individual, relational and institutional barriers is in line with recent evidence showing how stigma and adverse experiences in services influence continuity of care in the maternal–child continuum.^32^ From a programme perspective, the results also dialogue with evidence that loss and resumption of follow-up can be motivated by financial barriers, marital dynamics and (un)welcoming services. A recent qualitative study on loss and re-engagement in Option B+ describes this ‘back and forth’ motivated by economic factors, intimate relationships and care experiences.^10^ A critical finding is the tension between the desire to protect the baby and uncertainty (or conflicting messages) about infant feeding, ART and the risk of postnatal transmission. Recent population evidence shows relevant differences in breastfeeding duration by maternal HIV status, suggesting that mothers living with HIV may wean earlier, which reinforces the need for clinical and social support for optimal breastfeeding practices when recommended.^8^ In light of current WHO infant-feeding guidance, breastfeeding may be recommended when the mother is receiving effective ART, with sustained adherence support and appropriate monitoring.^6,7^ Therefore, the divergent infant-feeding guidance reported by participants may reflect individual clinical considerations or inconsistent counselling, reinforcing the need for clear, harmonised and non-stigmatising communication on infant feeding. The study indicates that trust in services is built primarily on confidentiality, respectful language and willingness to listen; conversely, lack of privacy, discriminatory attitudes and long waits degrade the experience and may deter women from following up. This pattern is consistent with recent literature that associates relational quality of care and climate of stigma with outcomes of retention and engagement in PMTCT.^10^ Recent evidence suggests that interventions to improve PMTCT service delivery and promote retention should address multiple steps of the PMTCT cascade, including maternal ART initiation, retention in PMTCT programmes, uptake of early infant diagnosis and infant outcomes.^33,34^ Continuous quality improvement interventions, with monitoring of engagement and organisational responses (e.g. screening for absences and proactive contact), may also be acceptable and feasible in PMTCT/Option B+ programmes.^4^ However, data from the present study indicate that ‘tracking absences’ is not sufficient if there is no stigma-free care environment. Retention strategies should combine organisational components (flows, privacy, mother–baby integration) with relational components (communication, welcoming, stigma reduction). The differences observed between younger and older women and between women with and without partners suggest that risk and needs are not homogeneous. Evidence on Option B+ retention identifies young age and non-disclosure as factors associated with non-retention,^12^ while qualitative evidence among young pregnant and postpartum women living with HIV shows that stigma, disclosure concerns and variable social support shape care and coping.^35^ Thus, the study supports differentiated approaches, including reinforced counselling and peer support for younger women, safe disclosure support for women afraid of abandonment or violence and social support mechanisms to mitigate economic and relational barriers. It is also important to consider that the narratives presented here predominantly reflect the experiences of women who remained at least partially connected to health services and who could be contacted and agreed to participate in the study. Women who were completely disengaged from PMTCT follow-up, who avoided institutional contact or who experienced greater social, geographic or economic vulnerability may be underrepresented. Their experiences could potentially involve more severe barriers to retention, lower continuity of care, greater mistrust of health services and more intense experiences of stigma or abandonment. In addition, all participants were recruited from a large tertiary referral maternity hospital in Luanda, a setting with greater technical capacity and specialised maternal–child HIV services than many primary health care facilities in Angola. Consequently, the findings should not be interpreted as representative of all postpartum women living with HIV in Angola, particularly those receiving care exclusively in peripheral or lower-resourced primary health care settings. The transferability of the findings should therefore be interpreted with caution and in relation to similar urban referral contexts. These findings reinforce the need to address psychosocial, relational and organisational determinants within PMTCT programmes, particularly during the postpartum period.

### Limitations

This study has limitations that should be considered when interpreting the findings. Firstly, it was conducted in a single urban tertiary referral maternity hospital in Luanda, with greater technical and organisational capacity than many peripheral or rural health facilities. Therefore, the findings may not be transferable to women receiving care in lower-resourced primary care settings or rural contexts. Secondly, data collection occurred within the institution where participants received care. Although privacy and confidentiality were ensured, this setting may have favoured socially desirable responses, particularly regarding adherence, relationships with health professionals and compliance with clinical guidance. Thirdly, the recruitment process may have introduced selection bias. Of 50 women contacted by telephone, 20 participated. Non-participation was related to non-response, explicit refusal, logistical difficulties, transport-related constraints and, in some cases, partner-related barriers. As a result, the sample may overrepresent women with greater availability, autonomy, service connection or logistical capacity while underrepresenting women who were disengaged from PMTCT care or experiencing greater social, economic or relational vulnerability. Fourthly, the interviews captured experiences up to 6 months postpartum, a period of heightened emotional and practical demands. Longitudinal studies could explore how perceptions, adherence and coping strategies evolve over time. Fifthly, the study focused only on postpartum women; the absence of perspectives from partners, family members and health professionals’ limits understanding of the wider relational and institutional dynamics shaping PMTCT experiences.

Despite these limitations, the study provides relevant qualitative evidence on emotional, social and organisational dimensions of postpartum PMTCT that are not captured by routine quantitative indicators.

### Implications for primary health care

Although this study was conducted in a tertiary referral maternity hospital, its findings are relevant to primary health care in Angola, where much antenatal, postnatal and infant follow-up care occurs. The barriers identified – stigma, inconsistent counselling, transport costs, partner-related constraints, limited psychosocial support and fragmented mother–baby follow-up – may be intensified in lower-resourced primary care settings.

Four implications emerge. Firstly, access to care should be strengthened through decentralised counselling, community-based follow-up, transport-sensitive appointment systems and stigma-sensitive reception procedures. Secondly, continuity of care requires clearer referral pathways from maternity hospitals to primary care, written appointment information, active follow-up of missed visits and integrated mother–baby appointments. Thirdly, person-centred care should recognise that women’s needs vary by age, partner status, disclosure concerns and emotional adaptation, requiring differentiated counselling and psychosocial support. Fourthly, coordination between levels of care should be improved through harmonised PMTCT protocols, shared mother–baby follow-up records and better communication between maternity, HIV and child health services.

## Conclusion

This study shows that postpartum prevention of vertical transmission of HIV is shaped not only by biomedical factors, such as ART adherence, infant feeding and follow-up, but also by emotional, relational and institutional determinants. Women’s experiences revealed that stigma, disclosure concerns, gender dynamics, quality of clinical communication and trust in health services influence continuity of care during the postpartum period. Motherhood emerged both as a source of vulnerability and as a strong motivation for treatment adherence and infant protection. The findings support the need for integrated, woman-centred and differentiated PMTCT care, combining consistent clinical monitoring, psychosocial support, safe disclosure counselling, stigma reduction and effective mother–baby follow-up. By foregrounding women’s voices, this study contributes context-specific qualitative evidence to inform improvements in postpartum PMTCT services in Angola and similar settings.
